# Utility of Narrow-Band Imaging during Intraoperative Colonoscopy for Colon Tumor Localization: A Report of Two Cases

**DOI:** 10.70352/scrj.cr.26-0091

**Published:** 2026-05-21

**Authors:** Mitsuki Nakazawa, Kaori Watanabe, Hiroyuki Asai, Shuhei Uehara, Akira Kato, Takuya Suzuki, Hajime Ushigome, Yushi Yamakawa, Hiroki Takahashi, Hiroyuki Sagawa, Takafumi Sato, Yoichi Matsuo, Akira Mitsui, Shuji Takiguchi

**Affiliations:** Department of Gastroenterological Surgery, Nagoya City University Graduate School of Medical Sciences, Nagoya, Aichi, Japan

**Keywords:** colonoscopy, narrow-band imaging, laparoscopic colorectal surgery

## Abstract

**INTRODUCTION:**

Accurate intraoperative localization of colonic tumors is essential to ensure appropriate resection margins, particularly in laparoscopic surgery where tactile feedback is limited. Intraoperative colonoscopy combined with narrow-band imaging (NBI) may facilitate precise delineation of tumor borders, but its clinical utility remains underreported.

**CASE PRESENTATION:**

We report 2 cases of cecal tumors in which intraoperative colonoscopy with NBI was employed to identify tumor margins and guide resection. The first case was a 79-year-old man with a positive fecal occult blood test, in whom colonoscopy revealed a 25-mm laterally spreading tumor (LST) in the cecum. During laparoscopic surgery, intraoperative colonoscopy with NBI clearly visualized the lesion, enabling cecal resection with negative histopathological margins. The second case was a 75-year-old woman referred for surgery following the detection of a 30-mm LST involving the appendiceal orifice. Intraoperative colonoscopy with NBI enabled accurate margin assessment, and the tumor was resected without residual disease. Histopathology confirmed tubulovillous adenoma with negative resection margins.

**CONCLUSIONS:**

Intraoperative colonoscopy with NBI was effective for identifying tumor borders and ensuring adequate resection margins in both cases. This approach may be a valuable adjunct in selected patients undergoing laparoscopic colorectal surgery, particularly for tumors located in anatomically complex regions such as the cecum or appendiceal orifice.

## Abbreviations

LST
: laterally spreading tumor
NBI
: narrow-band imaging
NIR
: near-infrared

## INTRODUCTION

In colorectal tumor resection, securing an adequate surgical resection margin is crucial to preventing local recurrence and improving long-term outcomes.^1,2)^ Laparoscopic and robot-assisted procedures have become the standard surgical approaches in recent years, offering favorable results due to their minimally invasive nature and high-definition visualization.^3,4)^ However, these approaches lack tactile feedback, which may lead to inadequate margins or inadvertent tumor injury.^5)^ To address this limitation, we previously reported an intraoperative localization method using NIR fluorescence imaging.^6)^ Although effective, this method requires specialized equipment such as the da Vinci Xi Surgical System (Intuitive Surgical, Sunnyvale, CA, USA), the 1688 AIM 4K Camera System (Stryker Japan K.K., Tokyo, Japan), or the VISERA ELITE II system (Olympus, Tokyo, Japan), limiting its accessibility and widespread adoption.

As an alternative, we focused on NBI, a modality that does not depend on specific laparoscopic systems. NBI was developed in Japan in 1999 and has been commercially available since 2007.^7)^ It is now widely incorporated into standard endoscopy units worldwide and is routinely used in gastrointestinal diagnostic practice, making it a more versatile and broadly accessible technology compared to NIR fluorescence. We present 2 cases in which intraoperative colonoscopy combined with NBI facilitated accurate tumor localization during cecal tumor resection, together with a brief literature review.

## CASE PRESENTATION

### Case 1

**Fig. 1:**
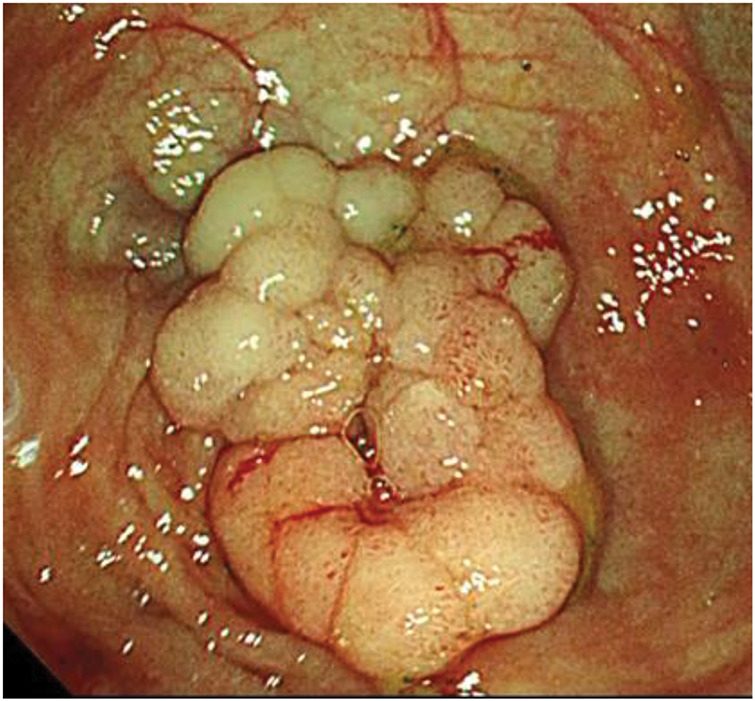
Preoperative colonoscopy for Case 1 showing a 25-mm LST in the cecum. LST, laterally spreading tumor

**Fig. 2:**
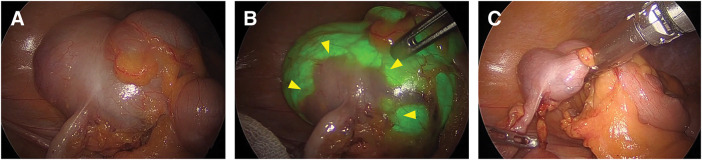
Intraoperative findings for Case 1. (**A**) Intraoperative view of the cecum under normal light. (**B**) Intraoperative NBI-assisted transillumination clearly delineating the tumor margins (yellow arrowheads). (**C**) Completion of cecal resection with an adequate surgical margin based on the identified borders. NBI, narrow-band imaging

**Fig. 3:**
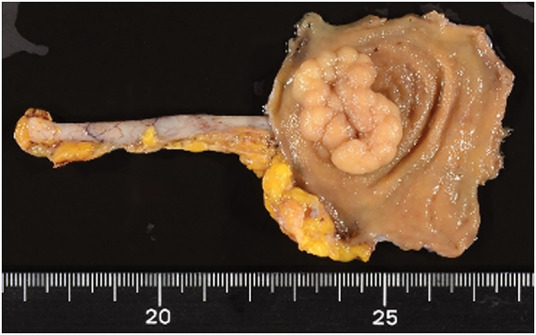
Resected specimen for Case 1 demonstrating negative surgical margins.

A 79-year-old male patient who had a positive fecal occult blood test during routine screening underwent colonoscopy, which identified a 25-mm LST in the cecum (**Fig. 1**). Histopathological analysis classified the polyp as a Group 3 tubular adenoma. He was subsequently referred to our institution for further management. Considering the tumor’s size and characteristics, surgical intervention was indicated, and laparoscopic cecal resection was performed. Intraoperative colonoscopy with NBI (Olympus) was employed to precisely delineate the tumor margins intraluminally, which clearly visualized the lesion’s boundaries from the serosal side (**Fig. 2A** and **2B**). Using a surgical stapling device, cecal resection was completed with an adequate margin based on the identified border (**Fig. 2C**). Histopathological analysis post-resection confirmed adenocarcinoma, with an invasion depth of M and negative resection margins (**Fig. 3**). The patient’s postoperative recovery was uneventful, and he was discharged on POD 6 without complications.

### Case 2

**Fig. 4:**
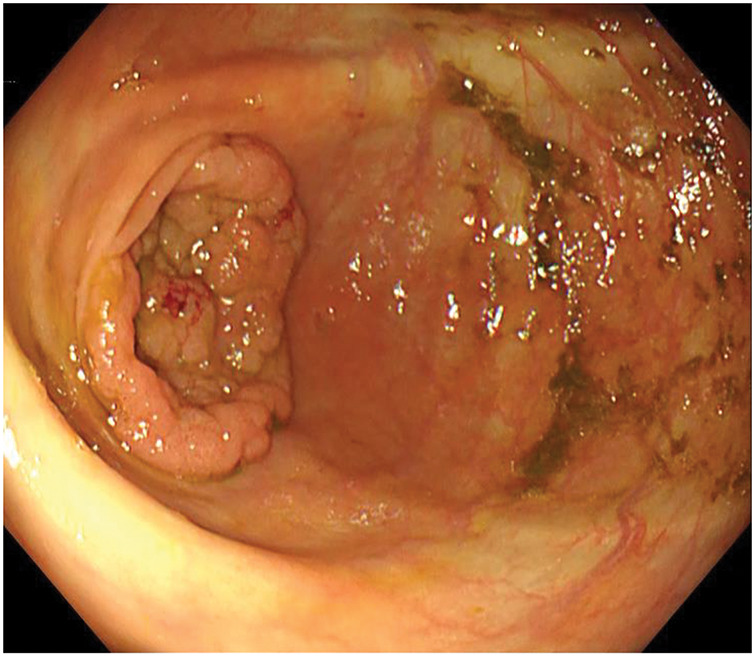
Preoperative colonoscopy for Case 2 revealing a 30-mm LST involving the appendiceal orifice in the cecum. LST, laterally spreading tumor

**Fig. 5:**
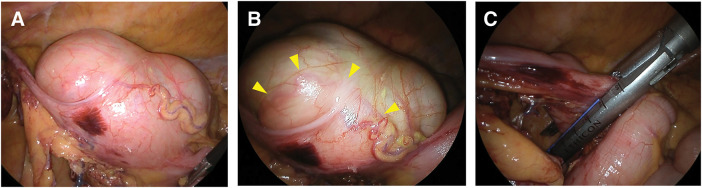
Intraoperative findings for Case 2. (**A**) Intraoperative normal light view of the cecum. (**B**) Visualization of the tumor outline using NBI transillumination (yellow arrowheads). (**C**) Resection with an adequate margin ensured by intraoperative localization. NBI, narrow-band imaging

**Fig. 6:**
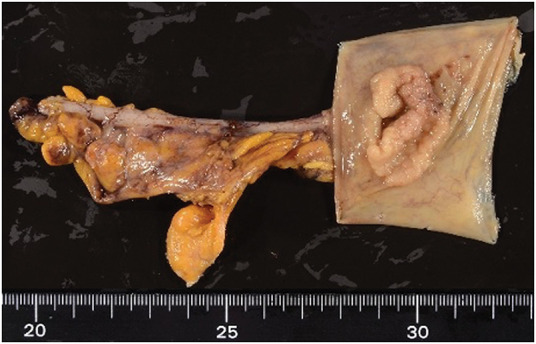
Resected specimen for Case 2 demonstrating negative margins.

A 75-year-old female patient presented to a referring physician with abdominal distension. Subsequent evaluation, including colonoscopy, identified a 30-mm LST in the cecum, leading to her referral to our institution. Repeat colonoscopy at our center confirmed a 30-mm LST located slightly distal to the appendiceal orifice within the cecum (**Fig. 4**). Histopathological analysis classified the polyp as a Group 3 tubular adenoma. No signs of distant metastasis or lymph node involvement were observed on CT. Subsequently, she underwent laparoscopic cecal resection. Intraoperatively, a colonoscope with NBI was utilized to precisely locate the tumor and ensure adequate resection margins (**Fig. 5A** and **5B**). The tumor was successfully resected with sufficient clearance from the identified boundary (**Fig. 5C**). Histopathological analysis confirmed the tumor as a tubulovillous adenoma with high-grade atypia. The surgical margin was negative (**Fig. 6**). The patient’s postoperative course was uneventful, and she was discharged on POD 7 without complications.

## DISCUSSION

In this study, tumor contours were visualized by irradiating the intestinal lumen with NBI light, a mode widely incorporated into standard endoscopes, and observing the tissue from the serosal side. To our knowledge, this is the first report describing intraoperative tumor localization from the serosal side using colonoscopy with NBI mode. NBI uses 2 specific wavelength ranges—blue light (390–445 nm) and green light (530–550 nm)—selected via specialized optical filters.^6)^ Given that oxidized hemoglobin has absorption peaks at 415 and 540 nm, NBI enhances the visualization of vascular architecture and mucosal microstructures. Clinically, this property is widely applied in endoscopic observation to detect avascular areas, vascular disruptions, and abnormal vessel morphology, thereby facilitating the early detection of malignancies.^8,9)^

**Fig. 7:**
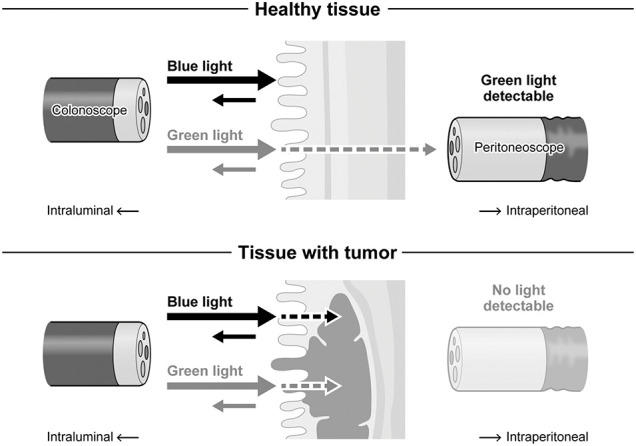
Schematic representation of the NBI-assisted transillumination mechanism. Colonoscopy emits blue and green light, with blue light absorbed by superficial vessels and green light penetrating deeper layers. NBI, narrow-band imaging

The 2 NBI wavelengths exhibit different behaviors across intestinal layers. Blue light, with its shorter wavelength, is predominantly absorbed and scattered by superficial mucosal vessels and rarely reaches the abdominal cavity. In contrast, green light penetrates deeper and can pass through normal tissue to reach the serosa, though partially attenuated by absorption and scattering within the submucosa.^10)^ However, in tumor tissue, the combination of increased thickness and hypervascularity enhances absorption of green light, blocking transmission. This differential behavior creates a contrast in transmitted green light between tumor and normal tissue, enabling visualization of tumor boundaries from the serosal surface (**Fig. 7**). Light transmission through biological tissue can be explained by the Lambert–Beer law (I = I₀·10^(–αcx)), where the transmitted intensity (I) decreases as tissue thickness (x) and cellular density (c) increase.^11)^ In tumors, both parameters are elevated due to tissue thickening and cellular proliferation, resulting in reduced light transmission. Furthermore, tumor angiogenesis increases hemoglobin concentration, which further reduces light penetration compared to normal tissue. These mechanisms likely allowed for the visualization of the tumor’s contour through the contrast between normal mucosa, through which NBI light penetrates, and tumor tissue, where light transmission is significantly attenuated.

From a clinical perspective, this technique may assist surgeons in precisely localizing tumors and ensuring adequate resection margins during laparoscopic colorectal surgery. The indications for this technique are limited, specifically to tumors that do not require lymph node dissection, can be treated with local resection, but are difficult to treat endoscopically, such as submucosal tumors and benign tumors/intramucosal carcinomas around the appendiceal opening. However, for these tumors, intraoperative tumor boundary clarification using NBI can facilitate the selection of safer resection lines and minimize the risk of positive margins and unintended tumor fragmentation. Although extracorporeal palpation following cecal mobilization is often a simple and effective method for tumor localization, flat lesions such as LSTs, or tumors located in anatomically complex areas, such as near the appendiceal orifice, may be difficult to identify by palpation alone. Furthermore, enlargement of the incision is necessary for palpation. In such cases, intraoperative colonoscopy using NBI may provide additional value because it allows for the identification of tumor boundaries from within the abdominal cavity. This method has potential applications beyond local resection of cecal tumors. For example, it may be advantageous in securing appropriate distal margins during rectal cancer surgery. Furthermore, its applicability extends beyond colorectal surgery to gastrectomy and duodenectomy, where accurate identification of tumor boundaries is crucial. In addition, because NBI systems are widely available in endoscopy units, intraoperative collaboration between surgeons and endoscopists could be enhanced, promoting a multidisciplinary approach that maximizes both safety and precision.

Despite these promising implications, several limitations should be noted. First, intraoperative colonoscopy requires an experienced endoscopist and may increase operative time. One case–control study has reported no significant difference in operative time between cases with and without intraoperative colonoscopy (e.g., 132 vs. 151 min, p = 0.5),^12)^ but in any case, synchronizing endoscopic and laparoscopic operations requires advanced skills. It is best not only to clamp the terminal ileum but also to temporarily release the pneumoperitoneum while inserting the colonoscope to the lesion site. Second, the efficacy of NBI-assisted transillumination is influenced by patient factors, such as bowel wall thickness, pericolic fat, and the presence of inflammation or fibrosis, all of which may attenuate light transmission and hinder tumor visualization. Reducing the light intensity of the laparoscope may make it easier to clarify the contrast of NBI light within the intestinal tract, but the usefulness of this method may be limited in obese patients or in tumors with significant fibrosis or inflammation, and so on. Third, only 2 discrete wavelengths of NBI were used in this study. To date, no reports have systematically evaluated the transmission properties of gastrointestinal tissues across a broader spectral range. Future research is expected to lead to clearer contouring by focusing on identifying the optimal wavelengths that maximize contrast between tumors and normal tissues using multispectral or hyperspectral imaging techniques. Finally, the oncological significance of this approach has not been fully validated. It remains unclear whether its use translates into improved outcomes, such as lower local recurrence rates or better disease-free survival. Through these efforts, intraoperative tumor localization using NBI may emerge as a practical, low-cost, and universally applicable modality that complements existing surgical technologies for safer oncologic resections.

## CONCLUSIONS

The method utilizing NBI in this report enables the visualization of colorectal tumor contours and facilitates safe resection with appropriate margins, independent of specific surgical equipment. Although the use of this technique should be carefully considered in selected cases, it holds potential for future application in various hybrid surgical procedures that combine laparoscopic and endoscopic procedures.

## DECLARATIONS

### Availability of data and materials

The datasets used and/or analyzed during the current study are available from the corresponding author on reasonable request.

## References

[1] Oka S, Tanaka S, Kajiwara Y, et al. Treatment decision for locally resected T1 colorectal carcinoma-verification of the Japanese Guideline Criteria for additional surgery based on long-term clinical outcomes. Am J Gastroenterol 2024; 119: 2019–27. 38345215 10.14309/ajg.0000000000002715 PMC11288396

[2] Detering R, Rutgers MLW, Bemelman WA, et al. Prognostic importance of circumferential resection margin in the era of evolving surgical and multidisciplinary treatment of rectal cancer: a systematic review and meta-analysis. Surgery 2021; 170: 412–31. 33838883 10.1016/j.surg.2021.02.029

[3] van der Pas MH, Haglind E, Cuesta MA, et al. Laparoscopic versus open surgery for rectal cancer (COLOR II): short-term outcomes of a randomised, phase 3 trial. Lancet Oncol 2013; 14: 210–8. 23395398 10.1016/S1470-2045(13)70016-0

[4] Feng Q, Yuan W, Li T, et al. Robotic versus laparoscopic surgery for middle and low rectal cancer (REAL): short-term outcomes of a multicentre randomised controlled trial. Lancet Gastroenterol Hepatol 2022; 7: 991–1004. 36087608 10.1016/S2468-1253(22)00248-5

[5] van der Meijden OA, Schijven MP. The value of haptic feedback in conventional and robot-assisted minimal invasive surgery and virtual reality training: a current review. Surg Endosc 2009; 23: 1180–90. 19118414 10.1007/s00464-008-0298-x PMC2686803

[6] Watanabe K, Takahashi H, Uehara S, et al. Visualization of cecal tumor by near-infrared laparoscopy and intraoperative colonoscopy. Surg Case Rep 2024; 10: 164. 38951358 10.1186/s40792-024-01964-0 PMC11217229

[7] Sano Y, Kobayashi M, Kozu T, et al. Development and clinical application of a narrow band imaging (NBI) system with built-in narrow-band RGB filters. Stomach Intest 2001; 36: 1283–7 (in Japanese).

[8] Sano Y, Tanaka S, Kudo SE, et al. Narrow-band imaging (NBI) magnifying endoscopic classification of colorectal tumors proposed by the Japan NBI Expert Team. Dig Endosc 2016; 28: 526–33. 26927367 10.1111/den.12644

[9] Iwatate M, Sano Y, Tanaka S, et al. Validation study for development of the Japan NBI Expert Team classification of colorectal lesions. Dig Endosc 2018; 30: 642–51. 29603399 10.1111/den.13065

[10] Gono K, Obi T, Yamaguchi M, et al. Appearance of enhanced tissue features in narrow-band endoscopic imaging. J Biomed Opt 2004; 9: 568–77. 15189095 10.1117/1.1695563

[11] Jacques SL. Optical properties of biological tissues: a review. Phys Med Biol 2013; 58: R37–61. 23666068 10.1088/0031-9155/58/11/R37

[12] Gorgun IE, Aytac E, Manilich E, et al. Intraoperative colonoscopy does not worsen the outcomes of laparoscopic colorectal surgery: a case-matched study. Surg Endosc 2013; 27: 3572–6. 23519496 10.1007/s00464-013-2928-1

